# Supplementary material for: Decreased Water Mobility Contributes To Increased α‐Synuclein Aggregation
Source: Angew Chem Int Ed Engl. 2023 Jan 12;62(7):e202212063. doi: 10.1002/anie.202212063 (PMC10107867; doi:10.1002/anie.202212063)
Supplement: Supplementary file 1 — Supporting Information [file ANIE-62-0-s001.pdf]

Angewandte Chemie
Eine Zeitschrift der Gesellschaft Deutscher Chemiker

Supporting Information

Decreased Water Mobility Contributes To Increased α-Synuclein Aggregation

*A. D. Stephens, J. Kölbel, R. Moons, C. W. Chung, M. T. Ruggiero, N. Mahmoudi, T. A. Shmool, T. M. McCoy, D. Nietlispach, A. F. Routh, F. Sobott, J. A. Zeitler, G. S. Kaminski Schierle**

Table of Contents

Table of Contents 2
1. Experimental Procedures 3
1.1 Purification of αSyn 3
1.2 Thioflavin-T based assays 3
1.3 Determination of remaining monomer concentration of αSyn after ThT-based assays using analytical size exclusion chromatography 3
1.4 Atomic Force Microscopy 3
1.5 Small Angle Neutron Scattering 4
1.6 Molecular Dynamics Simulations 4
1.7 Terahertz spectroscopy in liquid 5
1.8 Purification of αSyn for NMR analysis 5
1.9 Nuclear Magnetic Resonance Spectroscopy 6
1.10 Native nano-electrospray ionization mass spectrometry (nano-ESI-MS) and ion mobility (IM) 6
1.11 Terahertz time domain spectroscopy (THz-TDS) of solid samples 6
2. Results and Discussion 6
2.1 Supplementary Note 1: SANS data fitted with different models 6
2.2 Supplementary Note 2: MD simulations show solvated water and αSyn$_{72\text{-}78}$ peptide mobility is increased in the presence of CsI, but reduced in the presence of NaCl 7
2.3 Supplementary Note 3: Determining water absorption in the solvation shell in the absence of αSyn7
2.4 Supplementary Note 4: Nano-ESI-MS spectra 7
2.5 Supplementary Note 5: Nano-ESI-IM-MS spectra 7
2.6 Supplementary Note 6: Glass transition temperatures of solid state αSyn in CsI and NaCl determined by THz-TDS 7
Figure. S1. αSyn aggregation kinetics are enhanced in the presence of D_2O and for increasing concentrations of NaCl. 9
Figure. S2. Identification of flat and twisted αSyn fibril polymorphs by AFM. 10
Figure. S3. Representative analytical size exclusion chromatograph of remaining αSyn monomer after ThT-based assays. 11
Figure S4. Model fits to the SANS data using the Guinier-Porod model. 12
Table S1. Parameters of fitting SANS data presented in Figure 2 e,f. These results were obtained using a flexible cylinder model. The cylinder length fitted is not included as it is not detectable with the experimental q-range 13
Table S2. Parameters of fitting SANS data presented in Supplementary Figure 4 14
Table S3. Diffusion coefficient of the αSyn$_{72\text{-}78}$ peptide, solvation shell water and bulk water calculated using AIMD and classical MD simulations. 15
Table S4. Fit parameters for NaCl and CsI solutions with and without αSyn. 16
Figure S5. There is a greater relative difference of the molar absorption coefficient of water in NaCl compared to water in CsI. 17
Figure S6. Radial pair distribution functions extracted from AIMD simulations. 18
Figure S7. Absorption of water in the solvation shell independent of αSyn is lower for NaCl than CsI 19
Figure S8. Absorption of water in the solvation shell, excluding the protein itself, is compared to bulk absorption at 1 THz. 20
Figure S9. Detection of αSyn bound to NaCl and CsI at a 1:250 ratio. 21
Figure S10. αSyn is compact at lower charge states and extended at higher charge states in the absence of ions and in the presence of increasing concentrations of NaCl and CsI. 22
Figure S11. Intensity signal of ^{1}H and ^{15}N-labelled αSyn in 150 mM NaCl is slightly higher than in 150 mM CsI. 23
Table S5. Gradient, m, of the linear fit (y = mx+c) and respective glass transition temperatures determined using THz-TDS 24
Figure S12. Highly pure monomeric αSyn is isolated by liquid chromatography, as shown by a representative Coomassie blue stained gel and analytical RP. 25
References 26
Author Contributions 26
Data Availability 26

1. Experimental Procedures

1.1 Purification of αSyn

Human wild-type (WT) alpha-synuclein was expressed using plasmid pT7-7. The plasmid was heat shocked into *Escherichia coli* One Shot® BL21 STAR™ (DE3) (Invitrogen, Thermo Fisher Scientific, Cheshire, UK) and purified as previously described[1]. Recombinant αSyn was purified using ion exchange chromatography (IEX) in buffer A (10 mM Tris, 1 mM EDTA pH 8) against a linear gradient of buffer B (10 mM Tris, 1 mM EDTA, 0.5 M NaCl pH 8) on a HiPrep Q FF 16/10 anion exchange column (GE Healthcare, Uppsala, Sweden). αSyn was then dialysed into buffer C (1 M $(NH_4)_2SO_4$, 50 mM Bis-Tris pH 7) and further purified on a HiPrep Phenyl FF 16/10 (High Sub) hydrophobic interaction chromatography (HIC) column (GE Healthcare) and eluted against buffer D (50 mM Bis-Tris pH 7). Purification was performed on an ÄKTA Pure (GE Healthcare). αSyn was extensively dialysed against H_2O and used immediately for experiments or dialysed against 20 mM Tris pH 7.2 and concentrated using 10 k MWCO amicon centrifugal filtration devices (Merck KGaA, Darmstadt, Germany) and stored at −80 °C until use. αSyn in Tris was buffer exchanged into freshly prepared NaCl and CsI solutions before experiments using PD10 dialysis columns (GE Healthcare). Protein concentration was determined from the absorbance measurement at 280 nm on a Nanovue spectrometer using the extinction coefficient for αSyn of 5960 $M^{-1}cm^{-1}$. Protein purity was analysed using analytical reversed phase chromatography (aRP). Each purification batch was analysed using a Discovery BIO Wide Pore C18 column, 15cm x 4.6mm, 5µm, column with a guard cartridge (Supelco by Sigma-Aldrich, St. Louis, MO, USA) with a gradient of 95 % to 5 % H_2O + 0.1% trifluroacetic acid (TFA) or acetic acid and acetonitrile + 0.1% TFA or acetic acid at a flow-rate of 1 mL/min. The elution profile was monitored by UV absorption at 220 nm and 280 nm on an Agilent 1260 Infinity HPLC system (Agilent Technologies LDA, Santa Clara, USA) equipped with an autosampler and a diode-array detector. Protein purity fell between 88 - 95 % dependent on batch (Figure S12).

1.2 Thioflavin-T based assays

αSyn samples were buffer exchanged into 150 mM NaCl, 1.5M NaCl, 150 mM CsI or 1.5 M CsI in H_2O or D_2O prior to performing experiments using PD10 desalting columns (GE Healthcare). 10 µM freshly made ThT (abcam, Cambridge, UK) was added to 50 µL of 50 µM αSyn. All samples were loaded onto nonbinding, clear bottom, 365-well black plates (Greiner Bio-One GmbH, Kremsmünster, Austria). The plates were sealed with a SILVERseal aluminium microplate sealer (Grenier Bio-One GmbH). Fluorescence measurements were taken using a FLUOstar Omega plate reader (BMG LABTECH GmbH, Ortenberg, Germany). The plates were incubated at 37 °C with orbital shaking at 300 rpm for five minutes before each read every hour. Excitation was set at 440 nm with 20 flashes and the ThT fluorescence intensity was measured at 480 nm emission with a 1300 gain setting. ThT assays were repeated at least three times using at three or four wells for each condition. Data were normalised to the well with the maximum fluorescence intensity for each plate and the average was calculated for all experiments. Data are displayed with the rolling average from three experiments calculated using the program R (https://www.r-project.org/) with a 0.5 span. A linear trend line fitted along the exponential phase region of the ThT fluorescence curve was used to calculate the lag time (t_{lag}) at the intercept of the x axis, and the slope of the exponential phase (k) were calculated to show the elongation rate using equation 1.

$$y = kx - tlag \qquad (1)$$

1.3 Determination of remaining monomer concentration of αSyn after ThT-based assays using analytical size exclusion chromatography

To calculate the remaining αSyn monomer concentration in each well after ThT-based assays size exclusion chromatography performed on a high-pressure liquid chromatography (SEC) system was used. The contents of each well after the ThT-based assay were centrifuged at 21k x g for 20 minutes and the supernatant from each well was added to individual aliquots in the autosampler of the Agilent 1260 Infinity HPLC system (Agilent Technologies). 35 µL of each sample was injected onto an Advance Bio SEC column, 7.8 x 300 mm 130Å (Agilent Technologies) in 20 mM Tris pH 7.2 at 1 mL/min flow-rate. The elution profile was monitored by UV absorption at 220 and 280 nm. A calibration curve of known concentrations of αSyn was used to calculate the remaining monomer concentration of αSyn in each well. Six wells from two experiments were analysed for remaining monomer concentrations, the average value of each measurement is presented including the standard error of the mean (SEM).

1.4 Atomic Force Microscopy

The contents of wells from the ThT-based assays were centrifuged for 20 minutes at 21 k x g. 40 µL of supernatant was removed to leave 10 µL and remaining fibrils. The fibrils were resuspended and incubated on a freshly cleaved mica surface for 20 min. The mica

was washed three times in 18.2 Ω dH_2O to remove lose protein. Images were acquired in dH_2O using tapping mode on a BioScope Resolve (Bruker GmbH, Karlsruhe, Germany) using 'ScanAsyst-Fluid+' probes. 256 lines were acquired at a scan rate of 0.966 Hz per image with a field of view of 2-5 µm and for at least six fields of view. Images were adjusted for contrast and exported from NanoScope Analysis 8.2 software (Bruker).

1.5 Small Angle Neutron Scattering

Small-angle neutron scattering (SANS) measurements were performed on the SANS2D instrument at the ISIS Neutron and Muon Source (STFC Rutherford Appleton Laboratory, Didcot, UK). 6.25 mg of freeze-dried αSyn was dissolved in a salt solution containing 1.5 M NaCl or CsI in Milli-Q Ω18.2 water or pure deuterated water to give a final concentration of 434 µM. The protein solution was left stirring in a cooling cabinet for 0.5 h in H_2O salt solutions, whereas for D_2O salt solutions it was left for 1.5 h to allow for sufficient hydrogen/deuterium exchange. Samples were then loaded into quartz circular cells of 1 mm (H_2O samples) and 2 mm (D_2O samples) pathlength and measurements were made at room temperature. The protein solutions were also measured 15 to 19 hours after preparation.
An incident beam of 12 mm diameter, a wavelength range of 1.75–16.5 Å, and a setup of L_1 = 4 m; L_2 = 4 m, was used resulting in an effective range of scattering wave vector in equation 2,

$$q = \frac{4\pi}{\lambda} sin\left({}^{\theta}/_{2}\right),\ 0.005 \le q \le 0.7\ Å^{-1} \qquad (2)$$

where λ = the neutron wavelength and θ = the scattering angle. Raw scattering data are corrected for sample transmission, detector efficiency and solvent background scattering (as described in detail in[2]) using the Mantid Software, and then converted to absolute scattering cross section ($I(q)$ / cm^{-1}) using the scattering from a standard sample (comprising a solid blend of hydrogenous and perdeuterated polystyrene) in accordance with established procedures[3].
Modelling of the data was performed in SASView (http://www.sasview.org), using the Guinier–Porod model[4]. The scattering intensity, *I(q)*, is derived from independent contributions of the Guinier form in equation 3,

$$I(q) = \frac{G}{Q^s} \exp\left(\frac{-q^2 R_g^2}{3-s}\right)\ \ for\ q \le q_1 \qquad (3)$$

And the Porod form, in equation 4

$$I(q) = \frac{D}{Q^d}\ \ for\ q \ge q_1 \qquad (4)$$

where R_g is the the radius of gyration, d is the Porod exponent, and G and D are the Guinier and Porod scale factors, respectively. A dimensionality parameter (3 – s) is included in the Guinier form factor to help define non-spherical objects where s = 0 represents spheres or globules, s = 1 represents cylinders or rods and s = 2 represents lamellae or platelets.

1.6 Molecular Dynamics Simulations

Ab Initio Molecular Dynamics (AIMD)
The CP2k software package was used for all AIMD simulations, which incorporated three-dimensional periodic boundary conditions[5,6]. The simulations made use of the Perdew-Burke-Ernzerhof (PBE) density functional[7] coupled with the dispersion correction of Grimme[8,9]. The electronic wave functions were represented using the double-zeta DZVP basis set[5]. Simulations were performed within the canonical ensemble (NVT), with the temperature maintained at 300 K using a Nose-Hoover chain thermostat[10–12]. The crystal structure of seven amino acids, TGVTAVA residues 72-78 of αSyn, was taken from the protein database for simulations[13]. The initial model was generated by fully solvating the αSyn peptide with water molecules explicitly in a 64 nm^3 simulation box, totalling 2132 molecules. Ions were introduced to a concentration of 1.5 M to match the experiment. The simulations were equilibrated for 5 ps prior to performing the production MD runs over a 45 ps trajectory (50 ps total), with a time step of 1.0 fs. The convergence criteria for all AIMD simulations was set to a total energy of $\Delta E < 10^{-6}$ hartree. Diffusion coefficients were determined using the TRAVIS MD analyser[14]. Solvation shell water molecules were defined by defining a region of 1 nm around the peptide, with all water molecules within that region considered solvation shell waters.

Classical MD
The GROMACS software package was used for the classical MD simulations[15]. The TGVTAVA peptide structure was obtained from single-crystal X-ray diffraction experiments[13]. The peptide was solvated within a 125 nm^3 periodic box with water molecules (4264

total), and ions were introduced to a concentration of 1.5 M to match the experiments, as required. The interatomic forces were modelled using the OPLS-AA force field[16]. The structures were equilibrated within the NVT ensemble, followed by the NPT ensemble, each for 100 ps with a timestep of 2 fs. The temperature was controlled using a Nose-Hoover chain thermostat[10–12] and the pressure using the Parrinello-Rahman barostat[17]. Upon equilibration, production simulations were performed within the NVT ensemble (to match with the AIMD procedure), for a total of 10 ns with a timestep of 2 fs. Diffusion coefficients were determined using the TRAVIS MD analyser[14].The GROMACS simulations were performed in triplicate, with the average values from the three production simulations reported.

1.7 Terahertz spectroscopy in liquid

αSyn was dialysed extensively against H2O to remove salts after purification. The samples were snap frozen in liquid nitrogen and lyophilised using a LyoQuest 85 freeze-dryer (Telstar, Spain). The αSyn samples were resuspended at a concentration of 691.56 µM (10 mg/mL). 10 mM Tris pH 7.2 was added to the samples to aid reconstitution. Samples were reconstituted in the salts and sonicated for 10 s on and 10 s off for three times before THz measurements. The liquid was injected into a liquid cell with a path length of 100 µm. Reference measurements of buffer were performed using the same liquid cell. THz-TDS spectra were acquired using a commercial TeraPulse 4000 instrument with a spectral range of 0.2−2.7 THz (TeraView, Cambridge, UK). The temperature was kept constant at 294 K. The absorption coefficient of the liquid samples was calculated in the same way as that of solid samples. Measurements were repeated at least 5 times. Buffer and salt solutions were measured in 0.25 M increments for NaCl concentrations of 0.5 to 4 M, and for CsI concentrations of 0.25 to 2.5 M. αSyn in NaCl was measured at 2 M, as αSyn did not reconstitute successfully in concentrations below 2 M, and αSyn in CsI was measured at 1.25, 1.5, and 2 M each measurement was taken at least eight times. All spectra were subsequently divided by the salt concentration to obtain the molar absorption coefficient ε. ε was then fitted over frequencies with a linear function for samples containing NaCl. In samples containing CsI a spectral feature appeared at 0.7 THz and ε was therefore fitted with the sum of a power law and a Lorentzian to incorporate the spectral features, equation 5.

$$\varepsilon = \frac{\mathrm{A}}{1+\left(\frac{f-\mathrm{x}_1}{\mathrm{g}}\right)^2} + \mathrm{B}\cdot f^{\mathrm{a}} \qquad (5)$$

Where A is the peak intensity, g the half width at half maximum, x1 the centre frequency of the peak, f the frequency, and a and B are power law parameters. An offset was not observed in any measurement for which reason no absolute term is present.

The THz-TDS spectra of liquid αSyn in the two salts were deconvoluted to investigate the effects of the salt ions on the solvation shell. The solvation shell size of the single ions was based on the results obtained from the AIMD simulations. Because the absorption coefficients of the salts without protein present are known, absorption coefficients of the protein and its solvation shell can be calculated for different estimates of solvation shell sizes in equation 6.

$$\varepsilon = \varepsilon_{bulk} \cdot \frac{V_{bulk}}{V_0} + \varepsilon_{ps} \cdot \frac{V_{ps}}{V_0} = \varepsilon_{bulk} \cdot \frac{V_{bulk}}{V_0} + \varepsilon_p \cdot \frac{V_p}{V_0} + \varepsilon_s \cdot \frac{V_s}{V_0} \qquad (6)$$

1.8 Purification of αSyn for NMR analysis

E. coli were grown in isotope-enriched M9 minimal medium containing ^{15}N ammonium chloride similar our previous protocol[18]. To isolate expressed αSyn the cell pellets were resuspended in lysis buffer (10mM Tris-HCl pH 8, 1mM EDTA and EDTA-free complete protease inhibitor cocktail tablets (Roche, Basel, Switzerland), 0.2 mM phenylmethylsulfonyl fluoride (PMSF) and Pepstatin A) and lysed by sonication. The cell lysate was centrifuged at 22k x g for 30 min to remove cell debris and the supernatant was then heated for 20 min at 90 °C to precipitate the heat-sensitive proteins and subsequently centrifuged at 22k x g . Streptomycin sulfate 10mg/ml was added to the supernatant to precipitate DNA. The mixture was stirred for 15 min followed by centrifugation at 22k x g, then repeated. Ammonium sulfate 360 mg/ml was added to the supernatant precipitate the protein αSyn. The solution was stirred for 30 min and centrifuged again at 22k x g. The resulting pellet was resuspended in 25mM Tris-HCl, pH 7.7 and dialyzed overnight. The protein was purified by IEX on a HiPrep Q FF anion exchange column (GE Healthcare) and then further purified by SEC on a HiLoad 16/60 Superdex 75 prep grade column (GE Healthcare). All the fractions containing the monomeric protein were pooled together and concentrated using amicon 10 k MWCO centrifugal filtration devices (Merck). Protein purity was determined by aRP to be 88.6% (Supplementary Figure 8). αSyn was buffer exchanged into 5% D_2O and 95% H_2O using PD10 desalting columns (GE Healthcare). CsI and NaCl were added to a final concentration of 150 mM and 1.5 M just before performing the experiments.

1.9 Nuclear Magnetic Resonance Spectroscopy

NMR spectra were recorded as 2D ^{15}N HSQC at 298 K on a Bruker AV800 spectrometer (800 MHz ^{1}H) equipped with a 5 mm TXI HCN/z cryoprobe. Increase in salt concentration from 150 mM to 1.5 M resulted in an intrinsic signal intensity loss of 2.25x for NaCl and CsI, due to increased lossiness of the sample. Accordingly, spectra recorded at 1.5 M salt were multiplied by a factor of 2.25x prior to intensity analysis. Experiments were recorded with 2 scans per free induction decay with 150 and 1024 complex pairs in ^{15}N and ^{1}H, respectively.

1.10 Native nano-electrospray ionization mass spectrometry (nano-ESI-MS) and ion mobility (IM)

αSyn was buffer exchanged into 20 mM ammonium acetate (Sigma Aldrich, St. Louis, MO, USA) pH 7 using PD 10 columns (GE Healthcare) and diluted to a final concentration of 20 µM. NaCl (Acros Organics, New Jersey, USA) or CsI (Sigma Aldrich, St. Louis, MO, USA) were dissolved in water and added to the sample with a final concentration between 80 µM and 5 mM, which corresponds to a 1:4, 1:50 and 1:250 αSyn:Na/Cs ratio. The samples were incubated for ten minutes at room temperature before analysis. A Synapt G2 HDMS (Waters, Manchester, UK) was used to perform the nano-ESI (ion mobility-) mass spectrometry (nano-ESI-IM-MS) measurements. The results were analysed using Masslynx version 4.1 (Waters, Manchester, UK). Infusion of the samples into the mass spectrometer was performed using home-made gold-coated borosilicate capillaries. The main instrumental settings were: capillary voltage 1.5-1.8 kV; sampling cone 25 V; extraction cone 1 V; trap CE 4 V; transfer CE 0 V; trap bias 40 V. Gas pressures used throughout the instrument were: source 2.75 mbar; trap cell 2.3 x 10^{-2} mbar; IM cell 3.0 mbar; transfer cell 2.5 x 10^{-2} mbar. In mass spectra of the αSyn + CsI sample there are low intensity Na^{+} adducts remaining bound in spite of buffer exchange. Three experiments were performed using two purification batches of αSyn, there were no significant differences between experiments.

1.11 Terahertz time domain spectroscopy (THz-TDS) of solid samples

αSyn (2.2 mg/ml) was buffer exchanged into 1.5 M NaCl or 1.5 M CsI in H_2O using PD10 desalting columns. The samples were snap frozen in liquid nitrogen and lyophilised using a LyoQuest 85 freeze-dryer (Telstar, Spain). The samples were prepared into pellets between 300−600 µm in thickness as outlined previously[19]. This sample was sandwiched between the two z-cut quartz windows and sealed in the sample holder. The THz-TDS spectra were acquired using a commercial TeraPulse 4000 instrument across a spectral range of 0.2−2.7 THz (TeraView, Cambridge, UK). The experiments were conducted over a range of temperatures (100−390 K) using a continuous flow cryostat with liquid nitrogen as the cryogen (Janis ST-100, Wilmington MA, USA) as outlined previously[20]. To calculate the absorption coefficient and the refractive index of the sample, a modified method for extracting the optical constants from terahertz measurements based on the concept introduced by Duvillaret et al. was used[21,22]. The changes in sample dynamics were analysed by investigating the change in the absorption coefficient at a frequency of 1 THz and as a function of temperature. We have previously demonstrated that discontinuities in the temperature dependant absorption data in disordered materials can be observed that reflect change in the molecular dynamics of the sample. We have implemented a rigorous fitting routine based on statistical analysis to analyse the data and determine the transition points[20]. Three samples were measured and the error is displayed as s.d.

2. Results and Discussion

2.1 Supplementary Note 1: SANS data fitted with different models

The flexible cylinder model (Figure 2e,f, Table S1) gives a radius, R, a Kuhn length, b, that describes the stiffness of the fibril which can be related to shortness of fibril, and a length, L, [4]. A Kuhn length of 16 and 17 nm (Table 2) suggests the fibrils to be highly flexible. The fitting using the cylinder models to the scattering from αSyn in D_2O show some discrepancy especially at high *q*-range, indicating that other populations (monomers or/and oligomers) might influence the scattering curves or that fibrils in D_2O are not perfectly cylindrical. Instead, we used a flexible cylinder and sphere fitting (Figure 2g, Table 2 main text).
The Guinier-Porod model (Figure S4, Table S1) gives the radius, R, and a dimensionality parameter (3 – *s*) to help define non-spherical objects, where *s* = 0 represents spheres or globules, *s* = 1 represents cylinders or rods and *s* = 2 represents lamellae or platelets [41]. Both fits give an average radius of ~20 Å, but the radius increases over time for all αSyn samples apart from αSyn in CsI in H_2O. The Guinier-Porod model shows evidence of monomers in the high *q* region and presence of flexible rod-like structures where s = 1.2-1.4 (Table S1). The higher slope of the αSyn in NaCl samples in D_2O at low *q* indicates less rigid, i.e. longer aggregates, this is also observed in the cylinder model, where αSyn structures in NaCl are less rigid (Table 2).

2.2 Supplementary Note 2: MD simulations show solvated water and αSyn$_{72-78}$ peptide mobility is increased in the presence of CsI, but reduced in the presence of NaCl

To further probe the mechanisms behind the differences in aggregation rate of αSyn in the ionic solutions ab initio molecular dynamics (AIMD) and classical molecular dynamics simulations were used to elucidate the dynamics of the solvation shell(s), ions, as well as of αSyn[13] on femtosecond (fs) to picosecond (ps) timescales. The simulations were performed on a heptapeptide (TGVTAVA, residues 72-78) from the central region of αSyn, and solvated to the experimental density and ionic concentrations studied experimentally. The solvation shell waters were delineated from the bulk by defining a region within 1 nm of the peptide. The data highlight that the CsI system results in a significantly increased diffusion (i.e. further displacement from initial positions) of all of the components of the system, and yields in a self-diffusion constant of the αSyn$_{72-78}$ peptide that is significantly elevated compared to the pure water and NaCl models (Table S3, Figure 2a). This occurs through significant disruption of the water molecules near the coordination sphere of the Cs^+ cation. Due to its large size, a significant perturbation to the water geometries was observed. As a result, large-scale reorganisation of the water molecules occurs upon motion of Cs^+ ions which in turn leads to considerable shuttling of the water molecules. Given the strong intermolecular interactions as a result of dispersion, dipole-dipole, hydrogen bonding, and ion-dipole interactions between the water and αSyn$_{72-78}$ peptide molecules, this perturbation is coupled strongly to the αSyn$_{72-78}$ peptide molecule and affects the αSyn$_{72-78}$ peptide motions to a large spatial extent. It is important to note that in both the NaCl and CsI simulations, the number of ions within the first solvation shell of the αSyn$_{72-78}$ peptide remain consistent.

2.3 Supplementary Note 3: Determining water absorption in the solvation shell in the absence of αSyn

To investigate the absorption of the water in the solvation shell in the presence of the salts we first defined the parameters for salt-independent absorption, where the αSyn molar absorption coefficient (ε_P) is not influenced by the hydration with different salts. As ε_P is not known, the salt-independent absorption coefficient of water in the solvation shell was calculated based upon protein absorption defined between the upper boundary of 25 $cm^{-1}M^{-1}$, which is comparable to the absorption of αSyn in solid CsI at room temperature, and the lower boundary value, 1 $cm^{-1}M^{-1}$, around the limit of detection. Between the two boundaries the ratio of the absorption of water in the bulk and shell, independent of salt, is the same (Figure S7).

We subsequently investigated the influence of the salt on the water in the solvation shell, independent of the protein. The water shell containing NaCl absorbs less than the bulk above ε_P = 5 $cm^{-1}M^{-1}$, whereas the water shell containing CsI only absorbs less than the bulk above ε_P = 15 $cm^{-1}M^{-1}$ (Figure S7). If ε_p is higher than 15 $cm^{-1}M^{-1}$, both shells absorb less than the bulk, but the one containing NaCl even less so than the one including CsI. This shows that across a physiological range of protein absorption, ion and water mobility in the vicinity of αSyn are increased in CsI compared to NaCl. This is clearly observed at ε_P = 10 $cm^{-1}M^{-1}$ (shown by the circles in Figure S7), an intermediate protein absorption coefficient, where the water shell absorption in NaCl is reduced while increased in CsI.

2.4 Supplementary Note 4: Nano-ESI-MS spectra

It is likely that, at higher concentrations, even more Cs^+ and Na^+ ions are found to interact with αSyn, but increasing the salt concentrations in MS experiments leads to strong signal interference (Figure S9) Relatively low intensity Na^+ adducts can be observed in the αSyn + Cs^+ spectra (Figure 4a and Figure S9), the former are present in the purification buffer and are residually bound to αSyn after protein purification with a maximum of two Na^+ bound.

2.5 Supplementary Note 5: Nano-ESI-IM-MS spectra

The 8^+ charge state was chosen to reflect one of the most physiologically relevant charge state which resembles conformations present in solution elucidated by NMR [24] this state has multiple, clearly defined conformations. Higher charge states e.g. 11^+ and up correspond to more extended conformations with higher Coulombic repulsion (Figure S10).

2.6 Supplementary Note 6: Glass transition temperatures of solid state αSyn in CsI and NaCl determined by THz-TDS

Temperature ramping with THz-TDS showed that a solid state sample of αSyn and CsI had a secondary glass transition temperature at $T_{g,\beta}$ = 174 K while for αSyn and NaCl $T_{g,\beta}$ = 192 K. The glass transition at $T_{g,\beta}$ is associated with the onset of local mobility of the sample[25] and αSyn samples containing CsI become mobile at a lower temperature than αSyn samples containing NaCl (Figure 4b, Table S5). Furthermore, the linear gradient in the region $T < T_{g,\beta}$, associated with local mobility, or small scale mobility, is steeper for αSyn and CsI (m = 0.12 cm^{-1} K^{-1}) than αSyn and NaCl (m = 0.02 cm^{-1} K^{-1}) indicating that αSyn with CsI is more mobile than αSyn with NaCl. The αSyn and NaCl sample displays a second transition temperature, $T_{g,\alpha}$ = 254 K, which is associated with large scale mobility attributed to cooperative motions of the sample (Figure 4c, Table S5). No distinct $T_{g,\alpha}$ was observed for the αSyn sample

containing CsI indicating that there is sufficiently high mobility already present at lower temperatures. Hence, the cooperative motions gradually emerge at temperatures above $T_{g,\beta}$ instead of being associated with a defined transition point.

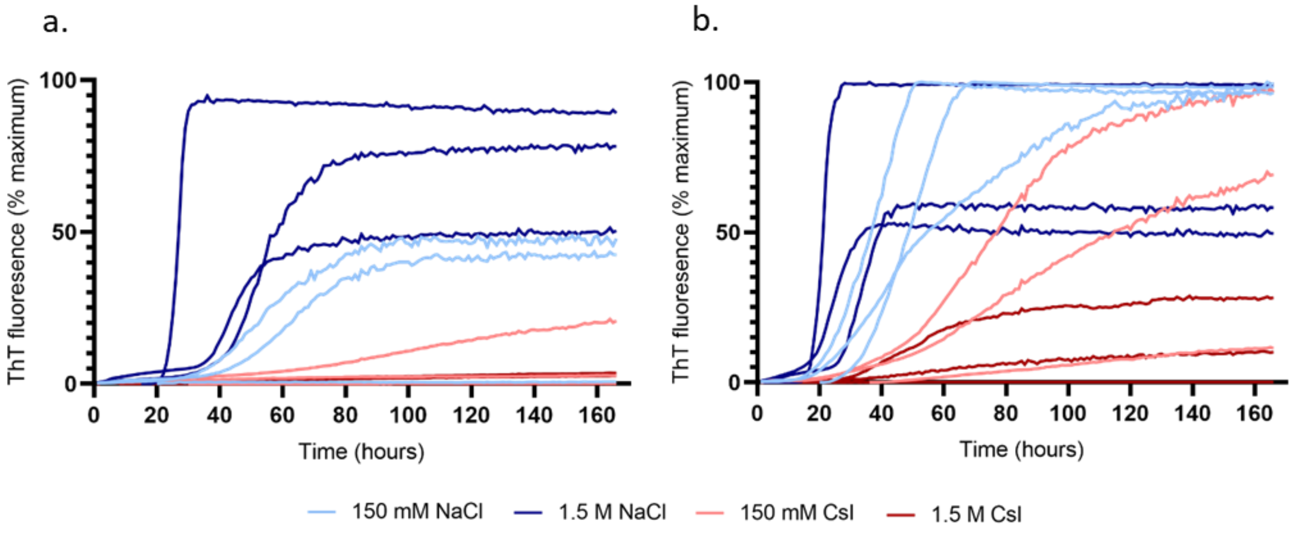

Figure. S1. αSyn aggregation kinetics are enhanced in the presence of D_2O and for increasing concentrations of NaCl.

αSyn aggregation kinetics were measured using a ThT fluorescence-based assay; 50 µM αSyn was incubated with 10 µM ThT in a 384 well plate with continuous orbital shaking for 160 hours in the presence of (a) H_2O and (b) D_2O with 150 mM NaCl (red), 1.5 M NaCl (brown), 150 mM CsI (blue), 1.5 M CsI (navy) and plotted as % maximum ThT fluorescence over time. Increased NaCl concentrations accelerated αSyn aggregation, while increased CsI concentrations decelerated αSyn aggregation. The aggregation rate in D_2O was enhanced compared to H_2O. Three plate repeats are shown for each condition, the data presented are the average of four wells per condition.

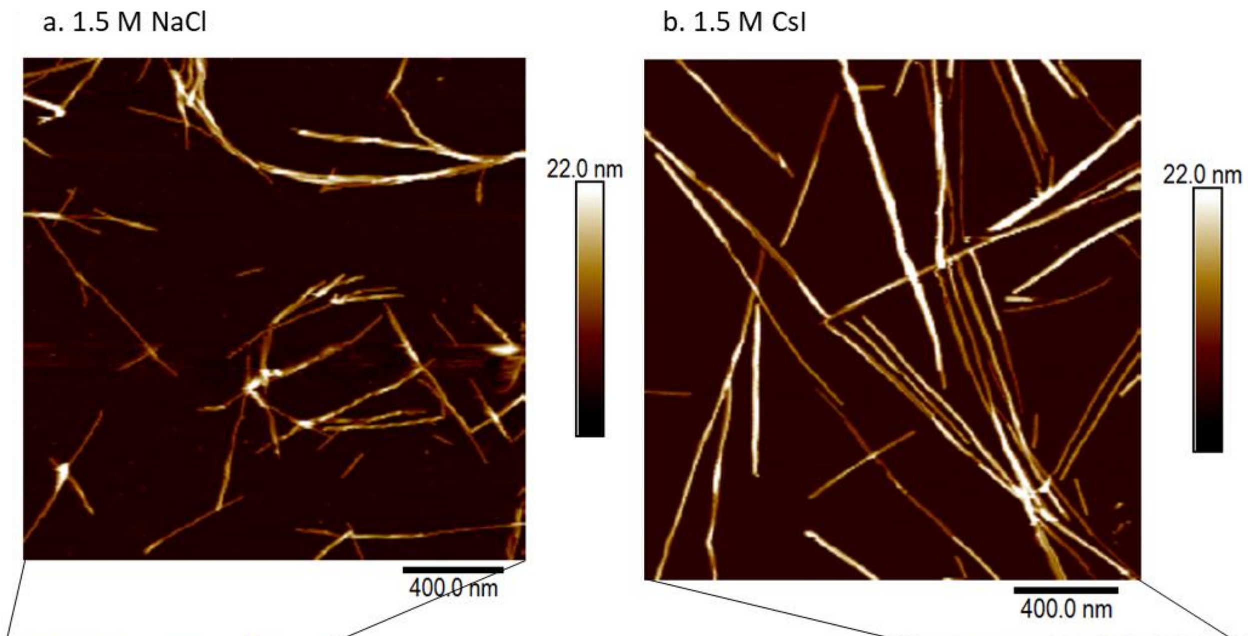

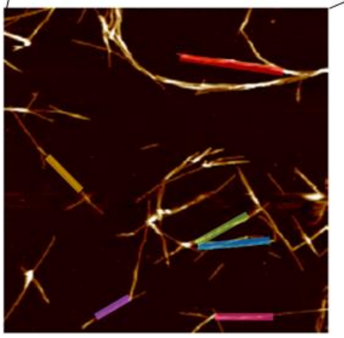

c.

Salt	Fibril Type	Average distance between peak (nm)
NaCl	Twist	70.5 ± 6.5
NaCl	Twist	78.5 ± 6.7
NaCl	Twist	56.9.8 ± 6.6
NaCl	Twist	109.3 0.5
NaCl	Flat	31.9 ± 11.9
NaCl	Flat	17.3 ± 4.0
CsI	Flat	31.5 ± 8.0
CsI	Flat	23.3 ± 8.3

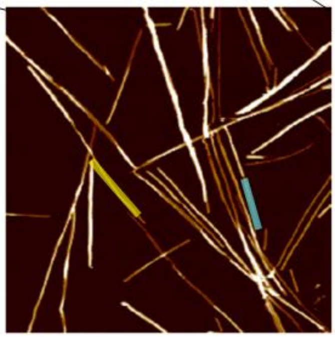

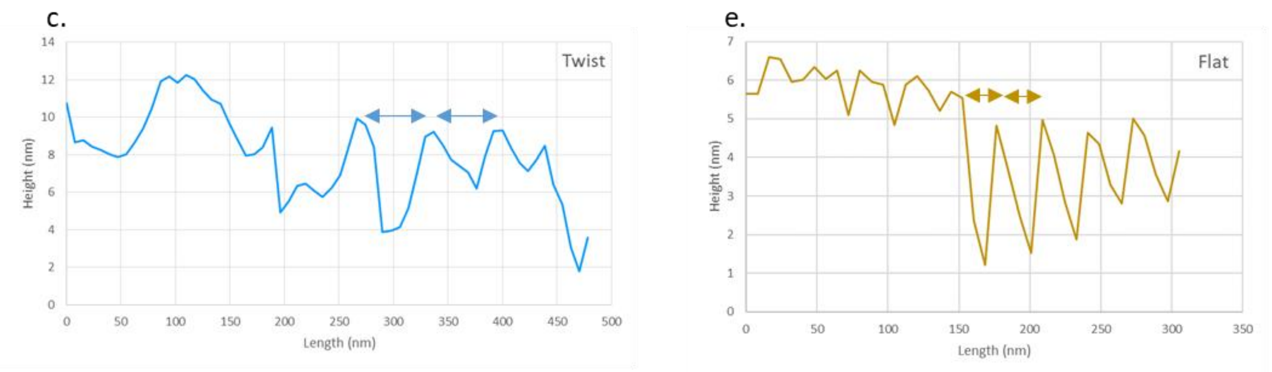

Figure. S2. Identification of flat and twisted αSyn fibril polymorphs by AFM.

αSyn fibrils formed during ThT-based assays were imaged on freshly cleaved mica and representative images are shown for different fibril polymorphs, ‘twisted’ containing a helical pitch and ‘flat’ with no visible helical pitch in (a.) 1.5 M NaCl in D_2O and (b.) flat only 1.5 M CsI in D_2O. Insets with coloured lines correspond to the colours in the table and show regions where fibril height was analysed. (c.) Table listing salt conditions, fibril type and distance measured between fibril peaks. Distances between peaks were calculated based on height profiles determined in the Nanoscope analysis software and are represented in (d.) for twisted fibrils with peak distances between 57-109 nm and (e.) flat fibrils with peak distances between 17 – 32 nm. The colours of the graph of peak heights in (d. + e.) correspond to the top blue twisted fibril and the bottom yellow flat fibril highlighted in table c and in the inserts of a and b.

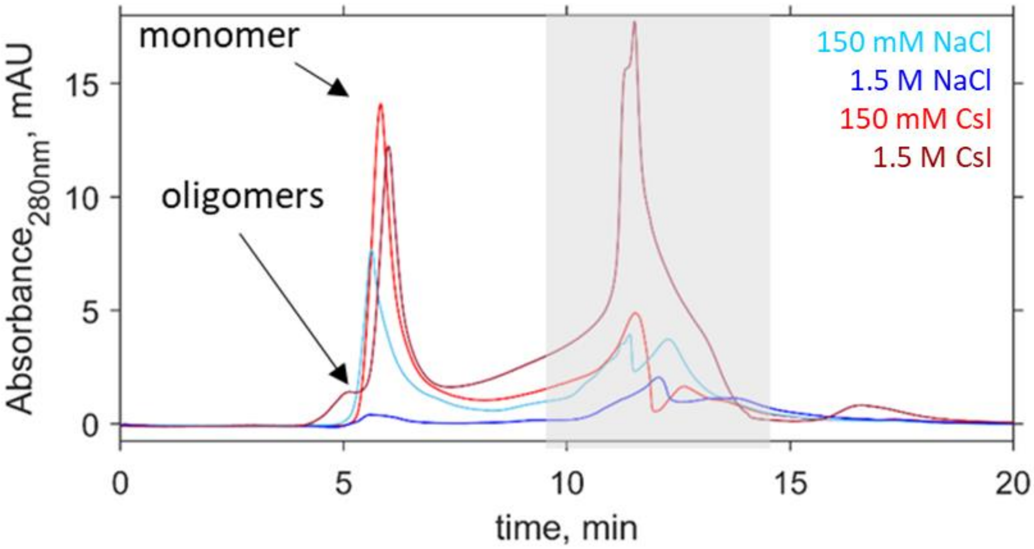

Figure S3. Representative analytical size exclusion chromatograph of remaining αSyn monomer after ThT-based assays.

The content of each well after the ThT-based assays was centrifuged and the remaining monomer analysed by analytical SEC-HPLC on an AdvanceBio 130Å column at a flow rate of 1 mL/min in 20 mM Tris pH 7.2 and monitored by absorbance at 280 nm. The area under the curve of the monomeric αSyn, which eluted ~ 5.2 minutes, was used to calculate the remaining monomer from known concentrations of αSyn. Representative chromatographs for αSyn in 150 mM NaCl (light blue, 1.5 M NaCl (dark blue), 150 mM CsI (red), 1.5 M CsI (dark red) are shown. Oligomeric species eluted before the monomer and can be detected in the 1.5 M CsI trace (dark red). Elution time for αSyn shifts slightly dependent on the salt and concentration present. The presence of salt is also detected in the grey region.

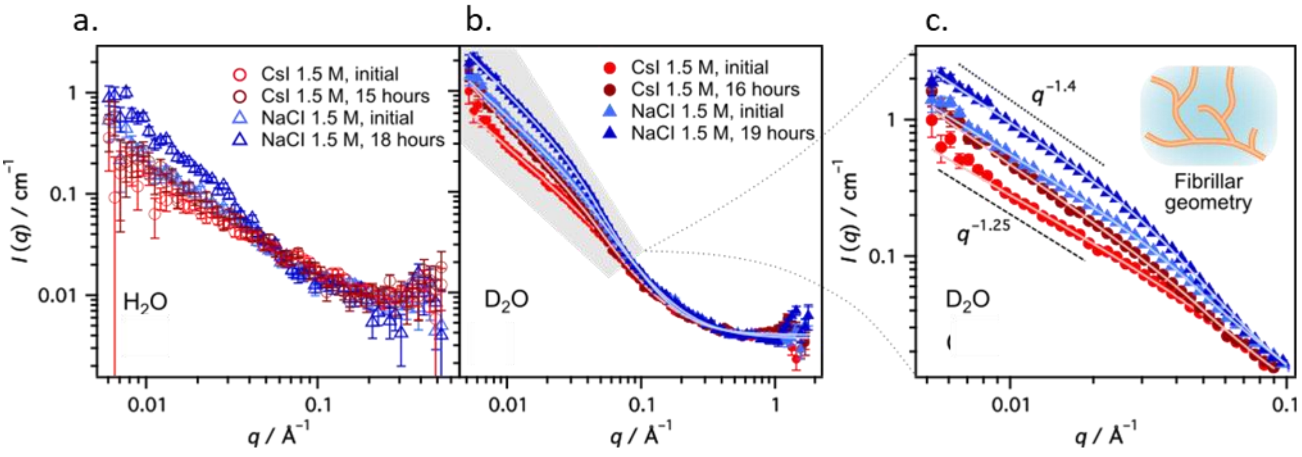

Figure S4. Model fits to the SANS data using the Guinier-Porod model.

SANS data for solutions of 434 µM αSyn in either 1.5 M NaCl or CsI in H_2O (a) and D_2O (b) at initial time points (light red and blue) compared to 15-19 hours later (dark red and blue). The αSyn in H_2O samples had high incoherent scattering, therefore the model was fit to D_2O data only. In c, solid symbols indicate experimental scattering data and solid lines represent the model fits. (c) Zoomed-in view of the low *q* region in b (shaded area) highlighting the differences in scattering intensity for each sample. Characteristic slopes are indicated with the data ($q^{-1.25}$ and $q^{-1.4}$), and the inset has been added to signify the fibrillar-type structure as determined by the modelling. Fitting values are shown in Table S2.

Table S1. Parameters of fitting SANS data presented in Figure 1e,f. These results were obtained using a flexible cylinder model. The cylinder length fitted is not included as it is not detectable with the experimental q-range

Solvent	Salt	Time	R (Å)	b (Å)	PDI of b	χ^2
H_2O	NaCl	Initial	21 ± 3	199 ± 116	0.95 ± 0.36	0.63
	NaCl	18 hours	27 ± 4	116 ± 61	0.48 ± 0.18	0.8
	CsI	Initial	20 ± 0.2	480 ± 42	0.11 ± 0.14	0.78
	CsI	15 hours	20 ± 0.9	468 ± 72	0.38 ± 0.17	0.8
D_2O	NaCl	Initial	20 ± 0.1	103 ± 14	0.65 ± 0.26	3.2
	NaCl	19 hours	22 ± 1.1	100 ± 5.7	0.99 ± 0.11	2.93
	CsI	Initial	20 ± 3.9	467 ± 111	0.91 ± 0.33	1.94
	CsI	16 hours	23 ± 4.2	409 ± 36	0.98 ± 0.38	2.65

Table S2. Parameters of fitting SANS data presented in Figure S4 using the Guinier-Porod model.

Solvent	Salt	Time	R (Å)	Dimension variable	Porod exponent
D_2O	NaCl	Initial	19.5	1.2	1.95
	NaCl	19 hours	23.1	1.3	2.28
D_2O	CsI	Initial	13.5	1.16	1.84
	CsI	16 hours	15.2	1.37	1.98

Table S3. Diffusion coefficient of the αSyn$_{72-78}$ peptide, solvation shell water and bulk water calculated using AIMD and classical MD simulations.

		**AIMD Diffusion Coefficient ($x10^{-10}$ m^2/s)**	**Classical MD Diffusion Coefficient ($x10^{-10}$ m^2/s)**
αSyn$_{72-78}$ peptide	Water	5.81	8.31 ± 0.21
	NaCl	6.71	9.25 ± 0.19
	CsI	15.43	14.46 ± 0.14
Solvation Shell Water	Water	19.99	24.13 ± 0.36
	NaCl	16.96	19.56 ± 0.41
	CsI	24.38	26.22 ± 0.32
Bulk Water	Water	23.60	33.45 ± 0.12
	NaCl	21.80	28.97 ± 0.16
	CsI	25.69	32.79 ± 0.11

Table S4. Fit parameters used for NaCl and CsI solutions with and without αSyn in THz data analysis.

**Parameter**	**NaCl solution**	**NaCl and αSyn solution**
m ($cm^{-1}M^{-1}THz^{-1}$)	18.2	15.4
n ($cm^{-1}M^{-1}$)	-3.7	-4.5

**Parameter**	**CsI solution**	**CsI and αSyn solution**
A ($cm^{-1}M^{-1}$)	27.8	23.2
x_1 (THz)	0.69	0.71
g (THz)	0.47	0.43
B (cm^{-1} M^{-1} THz^{-a})	19.5	17.0
a (a.u.)	1.13	1.25

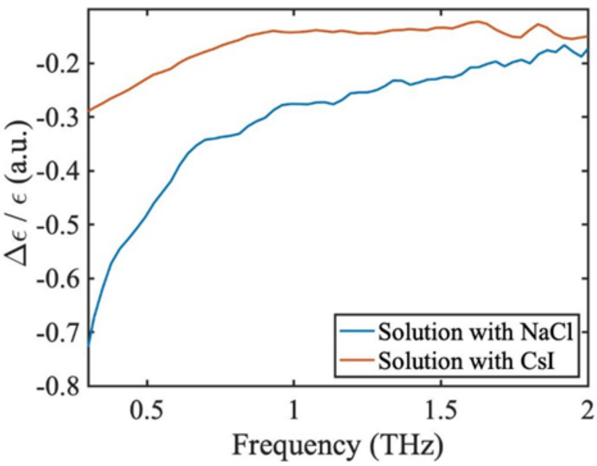

Figure S5. There is a greater relative difference of the molar absorption coefficient of water in NaCl compared to water in CsI.

The relative difference in the molar absorption coefficient of water, in the absence of spectral shape, between the different salts in the absence of αSyn (Δε) or in the presence of αSyn (ε) is shown for NaCl (blue) and CsI (orange). There is a greater difference in absorption of water in the presence of NaCl than CsI.

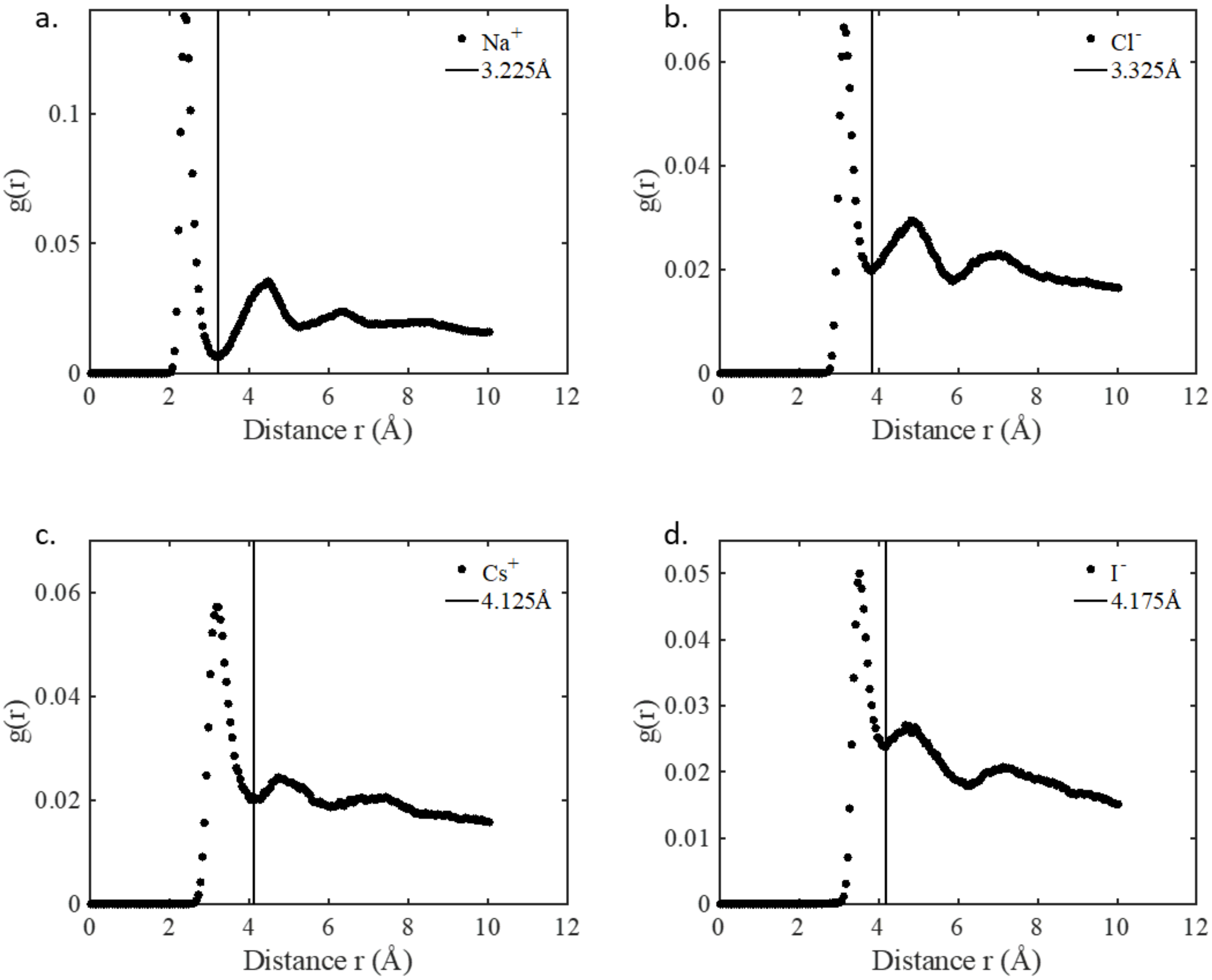

Figure S6. Radial pair distribution functions of Na^{+}, Cl^{-}, Cs^{+} and I^{-} extracted from AIMD simulations.

The first minima are used as a measure of the size of the solvation shell around different salt ions for THz data analysis (a) Na^{+}, (b) Cl^{-}, (c) Cs^{+}, (d) I^{-}. The horizontal line indicates the distance of solvation shell around each ion.

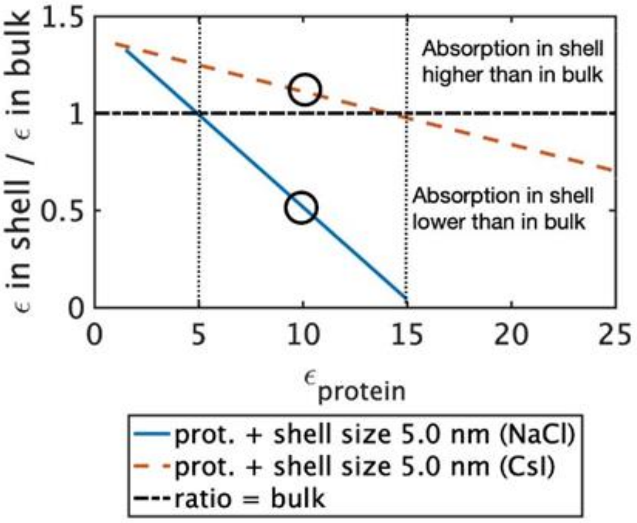

Figure S7. Absorption of water in the solvation shell, independent of αSyn, is lower for NaCl than CsI

Absorption of water in the solvation shell excluding the protein itself is compared to bulk water absorption at 1 THz. Representative ratio of the water absorption in the solvation shell compared to bulk water absorption for NaCl (blue line) and CsI (orange, dotted) for varying protein absorption (ε_P) between 1-25 $cm^{-1}M^{-1}$. The water in the solvation shell containing NaCl absorbs less than the bulk water (denoted by the black dashed horizontal line) above ε_P =5 $cm^{-1}M^{-1}$ (denoted by the black vertical dotted line at 5 $cm^{-1}M^{-1}$), while the water in the solvation shell containing CsI absorbs less than the bulk water above ε_P = 15 $cm^{-1}M^{-1}$ (denoted by the black dotted vertical line 15 $cm^{-1}M^{-1}$) at an ion shell size of 5 nm. At ε_P = 10 $cm^{-1}M^{-1}$, an intermediate protein absorption coefficient, the absorption of water in the solvation shell in the presence of NaCl is reduced while increased in CsI (shown by black circles). Other shell sizes are shown in Figure S8.

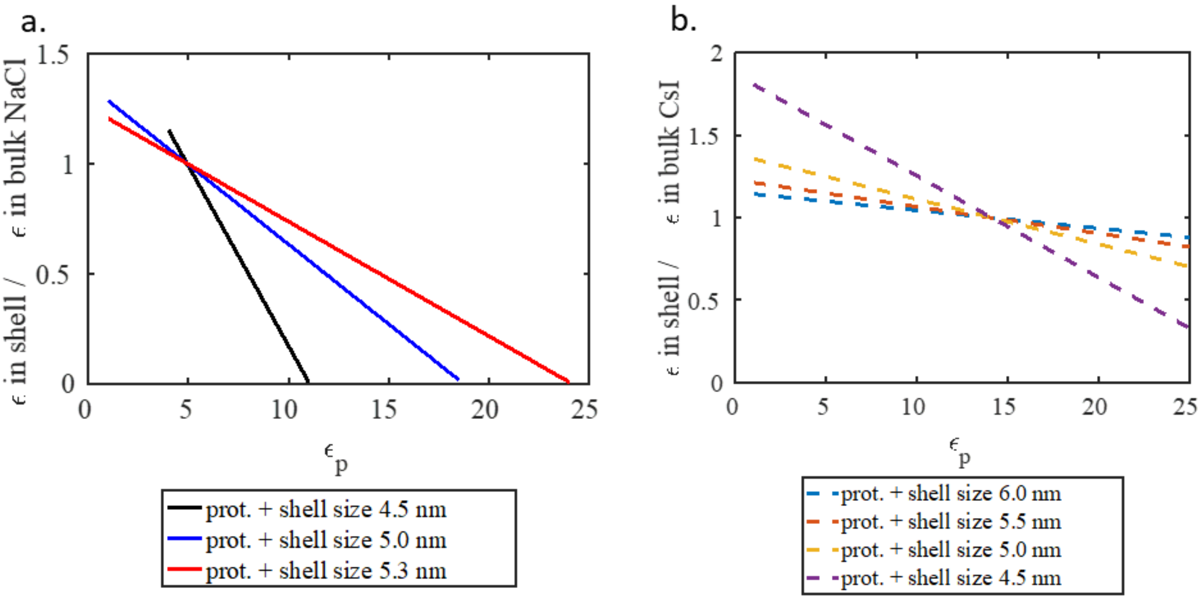

Figure S8. Absorption of water in the solvation shell, excluding the protein itself, is compared to bulk absorption at 1 THz.

Representative ratio of the absorption of water in the shell compared to bulk water absorption (a) in the presence of NaCl and (b) in the presence of CsI for varying protein absorption (ε_P) between 1-25 $cm^{-1}M^{-1}$. The water in the solvation shell in the presence of NaCl absorbs less than the bulk above ε_P =5 $cm^{-1}M^{-1}$, while the water in the solvation shell in the presence of CsI absorbs less than the bulk above ε_P = 15 $cm^{-1}M^{-1}$.

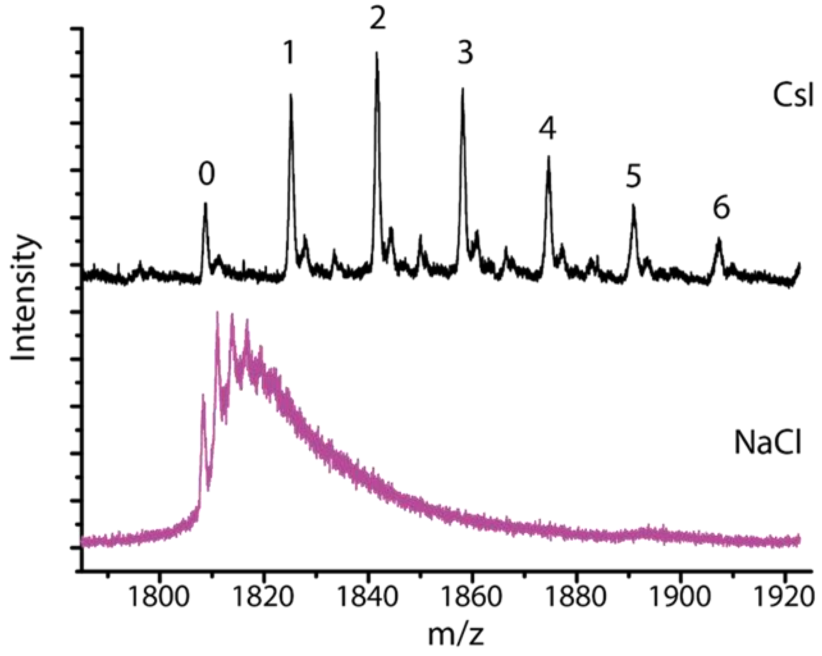

Figure S9. Detection of αSyn bound to NaCl and CsI at a 1:250 ratio.

Nano-ESI- MS spectra in positive-ion mode for a titration of 20 µM αSyn in 5 mM NaCl (purple) and 5 mM CsI (black). There are more observed ions bound to αSyn at 5 mM compared to 1 mM (Figure 4 main text), but for NaCl the resolution becomes distorted.

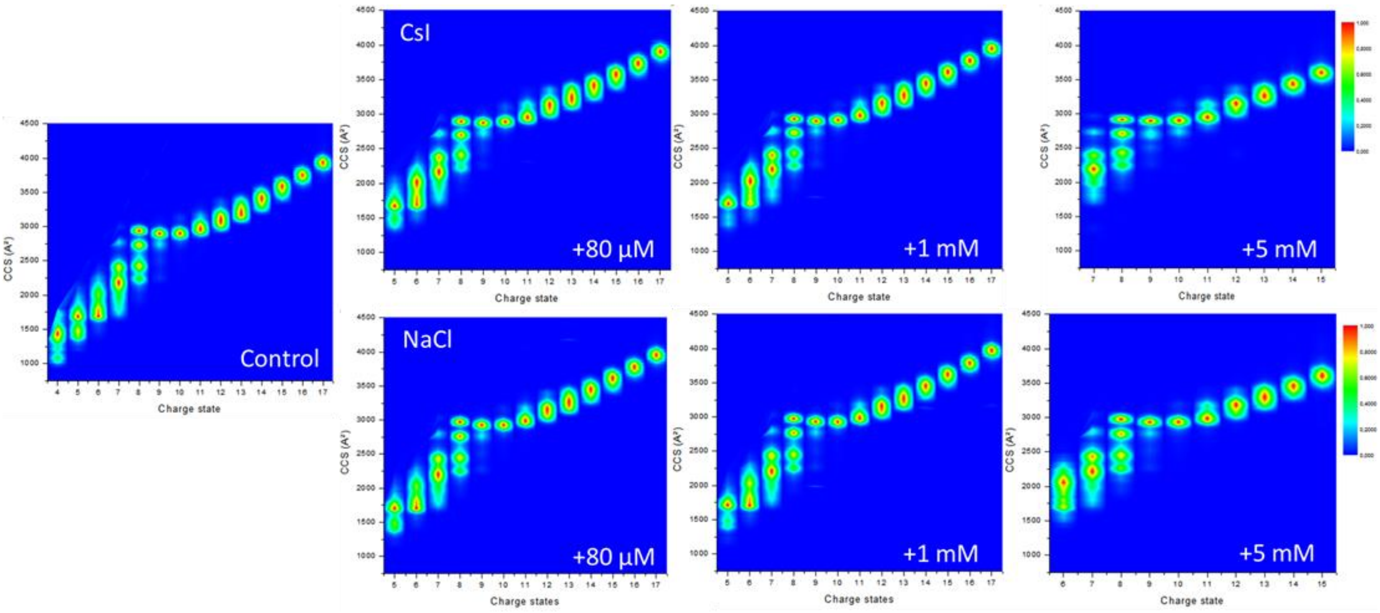

Figure S10. αSyn is compact at lower charge states and extended at higher charge states in the absence of ions and in the presence of increasing concentrations of NaCl and CsI.

Nano-ESI-IM-MS spectrum of αSyn at all charge states detected. αSyn in 50 mM ammonium sulphate with no salts (Control) and in the presence of 80 µM, 1 mM or 5 mM CsI and NaCl. Higher CCS values show more extended structures and extended structures are favoured at higher charge states. Shown are representative CCS plots from three injections of αSyn.

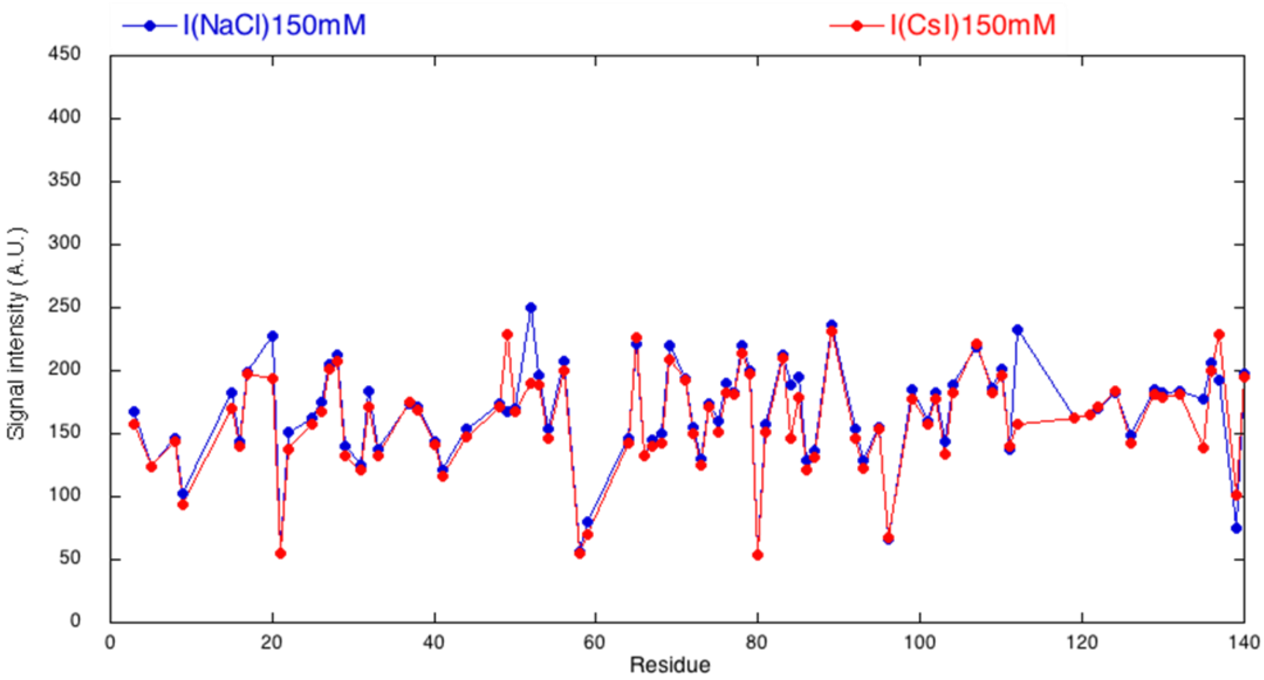

Figure S11. Intensity signal of ^{1}H and ^{15}N-labelled αSyn in 150 mM NaCl is slightly higher than in 150 mM CsI.

HSQC NMR spectroscopy was used to measure the intensity of 150 µM ^{1}H and ^{15}N-labelled αSyn in 95% H_2O, 5% D_2O (vol/vol) in 150 mM CsI and NaCl. Each residue covered is represented by a spot. The signal intensity of αSyn in 150 mM NaCl is similar, but marginally high than in CsI, indicating less mobility in NaCl.

Table S5. Gradient, m, of the linear fit (y = mx+c) and respective glass transition temperatures, $T_{g,\beta}$ and $T_{g,\alpha}$, determined using THz-TDS

Sample	Region 1 (cm^{-1} K^{-1})	Region 2 (cm^{-1} K^{-1})	Region 3 (cm^{-1} K^{-1})	$T_{g,\beta}$ (K)	$T_{g,\alpha}$ (K)
αSyn + CsI	0.12 ± 0.01	0.067 ± 0.001	-	174	-
αSyn + NaCl	0.02 ± 0.01	0.035 ± 0.001	0.059 ± 0.01	192	254

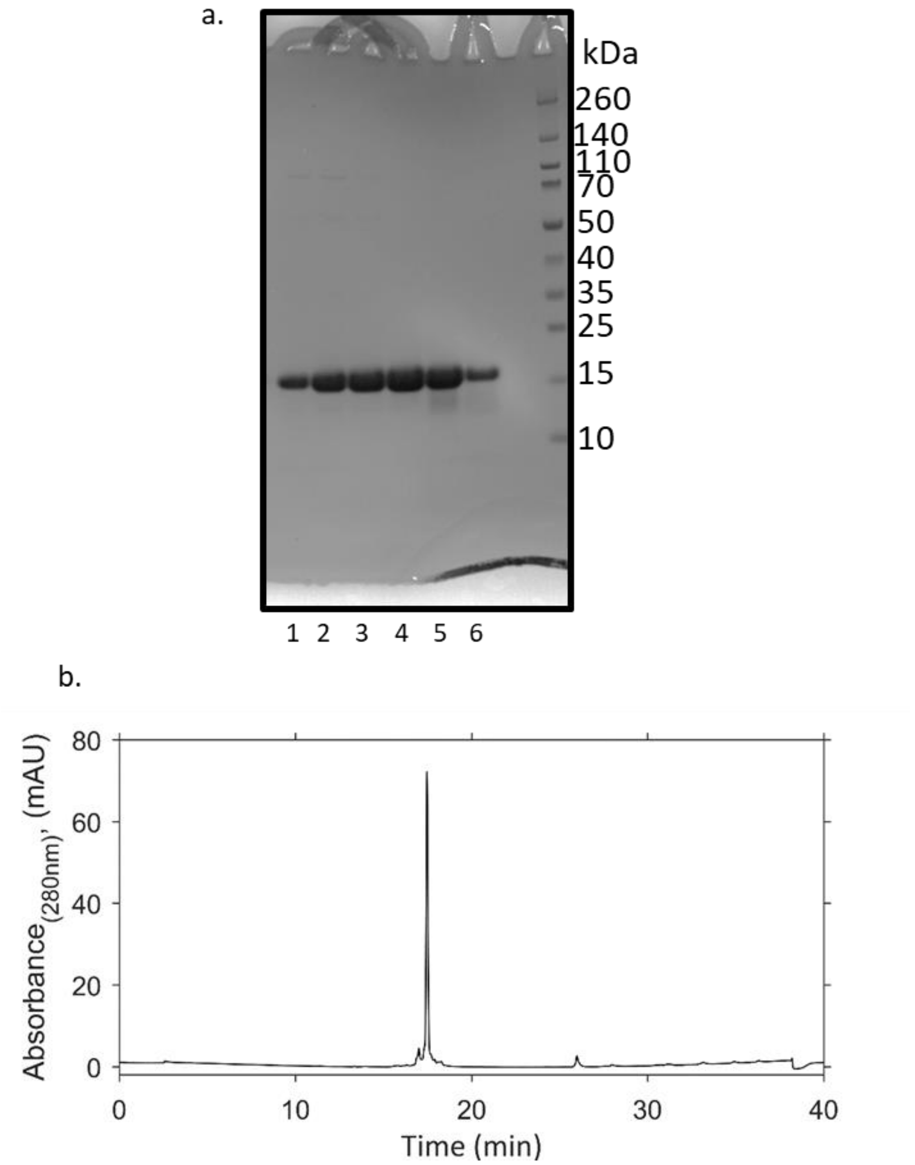

Figure S12. Highly pure monomeric αSyn is isolated by liquid chromatography, as shown by a representative Coomassie blue stained gel and analytical RP.

(a) ^{15}N-labelled αSyn after gel filtration, lanes 1-4 were used in experiments as lanes 5-6 contained degradation products. (b) 50 µL ^{15}N-labelled αSyn was analysed by analytical RP-HPLC on a Discovery Bio Wide Pore C18-5 column and eluted using a gradient of 5% acetonitrile + 0.1% acetic acid to 95% acetonitrile + 0.1% acetic acid with H_2O + 0.1% acetic acid over 40 minutes at 1 ml/min. Percentage purity of αSyn was 88.6% based on absorbance at 280 nm.

References

[1] A. D. Stephens, D. Matak-Vinkovic, G. S. Kaminski Schierle, *Biochemistry* **2020**, DOI 10.1101/2020.05.13.093286.
[2] R. K. Heenan, J. Penfold, S. M. King, *SANS at Pulsed Neutron Sources: Present and Future Prospectst*, **1997**.
[3] F. Carsughi, D. Bellmann, J. Kulda, M. Magnani, M. Stefanon, *Absolute Calibration of Small-Angle Neutron Scattering Data of a Double-Crystal Diffractometer*, **1997**.
[4] B. Hammouda, *J. Appl. Cryst* **2010**, *43*, 716–719.
[5] J. VandeVondele, J. Hutter, *J. Chem. Phys.* **2007**, *127*, 114105.
[6] J. VandeVondele, M. Krack, F. Mohamed, M. Parrinello, T. Chassaing, J. Hutter, *Comput. Phys. Commun.* **2005**, *167*, 103–128.
[7] J. P. Perdew, K. Burke, M. Ernzerhof, *Phys. Rev. Lett.* **1996**, *77*, 3865–3868.
[8] S. Grimme, J. Antony, S. Ehrlich, H. Krieg, *J. Chem. Phys.* **2010**, *132*, 154104.
[9] S. Grimme, S. Ehrlich, L. Goerigk, *J. Comput. Chem.* **2011**, *32*, 1456–1465.
[10] S. Nosé, *J. Chem. Phys.* **1984**, *81*, 511–519.
[11] S. Nosé, *Mol. Phys.* **1984**, *52*, 255–268.
[12] G. J. Martyna, M. L. Klein, M. Tuckerman, *J. Chem. Phys.* **1992**, *97*, 2635–2643.
[13] D. Li, E. M. Jones, M. R. Sawaya, H. Furukawa, F. Luo, M. Ivanova, S. A. Sievers, W. Wang, O. M. Yaghi, C. Liu, D. S. Eisenberg, *J. Am. Chem. Soc.* **2014**, *136*, 18044–18051.
[14] M. Brehm, M. Thomas, S. Gehrke, B. Kirchner, *J. Chem. Phys.* **2020**, *152*, 164105.
[15] S. Pronk, S. Páll, R. Schulz, P. Larsson, P. Bjelkmar, R. Apostolov, M. R. Shirts, J. C. Smith, P. M. Kasson, D. Van Der Spoel, B. Hess, E. Lindahl, *Bioinformatics* **2013**, *29*, 845–854.
[16] M. J. Robertson, J. Tirado-Rives, W. L. Jorgensen, *J. Chem. Theory Comput.* **2015**, *11*, 3499–3509.
[17] M. Parrinello, A. Rahman, *Phys. Rev. Lett.* **1980**, *45*, 1196–1199.
[18] G. Fusco, T. Pape, A. D. Stephens, P. Mahou, A. R. Costa, C. F. Kaminski, G. S. Kaminski Schierle, M. Vendruscolo, G. Veglia, C. M. Dobson, A. De Simone, *Nat. Commun.* **2016**, *7*, 12563.
[19] T. A. Shmool, P. J. Hooper, G. S. Kaminski Schierle, C. F. van der Walle, J. A. Zeitler, *Pharmaceutics* **2019**, *11*, 291.
[20] T. A. Shmool, J. A. Zeitler, *Polym. Chem.* **2019**, *10*, 351–361.
[21] L. Duvillaret, F. Garet, J.-L. Coutaz, *IEEE J. Sel. Top. Quantum Electron.* **1996**, *2*, 739–746.
[22] J. Sibik, J. A. Zeitler, *Philos. Mag.* **2016**, *96*, 842–853.
[23] J. Schindelin, I. Arganda-Carreras, E. Frise, V. Kaynig, M. Longair, T. Pietzsch, S. Preibisch, C. Rueden, S. Saalfeld, B. Schmid, J. Y. Tinevez, D. J. White, V. Hartenstein, K. Eliceiri, P. Tomancak, A. Cardona, *Nat. Methods* **2012**, *9*, 676–682.
[24] J. R. Allison, R. C. Rivers, J. C. Christodoulou, M. Vendruscolo, C. M. Dobson, **2014**, *53*, 7183.
[25] M. T. Ruggiero, M. Krynski, E. O. Kissi, J. Sibik, D. Markl, N. Y. Tan, D. Arslanov, W. Van Der Zande, B. Redlich, T. M. Korter, H. Grohganz, K. Löbmann, T. Rades, S. R. Elliott, J. A. Zeitler, *Phys. Chem. Chem. Phys.* **2017**, *19*, 30039–30047.
[26] C. Huang, G. Ren, H. Zhou, C. Wang, *Protein Expr. Purif.* **2005**, *42*, 173–177.

Author Contributions

A.D.S. and G.S.K.S. conceived the project and designed experiments. A.D.S purified protein for all experiments, performed kinetic aggregation assays and AFM. N.M. performed SANS experiments, N.M and T.M analysed SANS data. MD simulations were performed by M.T.R. J.K. performed solution THz-TDS experiments. R.M. performed nano-ESI-MS and nano-ESI-IM-MS experiments. D.N. performed NMR experiments. T.A.S. performed solid THz-TDS experiments. C.W.C performed experiments not in the final manuscript. A.F.R, F.S. and J.A.Z provided resources and scientific discussion. All authors contributed to editing the manuscript and have given approval to the final version of the manuscript.

Data Availability

Raw data is available at the University of Cambridge Repository (DOI: https://doi.org/10.17863/CAM.63587). The SANS experiment at the ISIS Neutron and Muon Source was allocated under the beamtime XB1890203 (DOI: http://doi.org/10.5286/ISIS.E.RB1890203-1).
